# Ascending bacterial optic neuritis and meningoencephalitis following tooth extraction in a cat

**DOI:** 10.1186/s12917-025-05024-z

**Published:** 2025-12-15

**Authors:** Solveig Reeh, Ana Cristina Piroth, Manon Mikic, Svenja Becker, Melanie Stoff, Andreas Beineke, Jutta Verspohl, Claudia Busse, Jasmin Neßler, Andrea Tipold

**Affiliations:** 1 Department of Small Animal Internal Medicine and Surgery, University of Veterinary Medicine Hannover, Foundation, Buenteweg 9, Hannover, 30559 Germany; 2 Institute of Pathology, University of Veterinary Medicine Hannover, Foundation, Buenteweg 17, Hannover, 30559 Germany; 3 Institute of Microbiology, University of Veterinary Medicine Hannover, Foundation, Bischofsholer Damm, Hannover, 30173 Germany

**Keywords:** Encephalitis, Optic neuritis, Tooth extraction, Feline

## Abstract

**Background:**

Dental procedures in cats are routinely performed, with complications rarely reported. However, when complications do occur, they can be severe, potentially resulting in penetrating ocular trauma, often leading to vision loss and subsequent enucleation. This report describes retrobulbar abscessation, ascending bacterial optic neuritis, and bacterial meningoencephalitis as a complication following routine tooth extraction in a cat.

**Case presentation:**

A 12-year-old cat was presented six days after a routine dental procedure due to left-sided eye swelling, inappetence, and lethargy. General and ophthalmic examination revealed absent vision, hyphema, and exophthalmos of the left eye, suspected to be secondary to ocular trauma. During hospitalization, the cat’s mental status deteriorated, and a generalized epileptic seizure was observed. Due to the poor prognosis, the owners elected euthanasia.

A postmortem MRI of the skull revealed retrobulbar and optic nerve inflammation, meningoencephalitis, and a suspected intracranial abscess potentially associated with the recent dental extraction. Necropsy confirmed a retrobulbar abscess, ascending bacterial optic neuritis, and bacterial meningoencephalitis.

**Conclusion:**

Bacterial meningitis and encephalitis should be considered as differential diagnoses in cats presenting with ocular changes and intracranial neurologic signs following routine dental procedures.

## Background

Tooth extractions are the most frequently performed oral surgeries in dogs and cats. Minor complications from these procedures have been described, ranging from root fractures during extraction to less commonly reported severe complications such as mandibular fractures, fistula formation, or, rarely, orbital penetration [1].

**Fig. 1:**
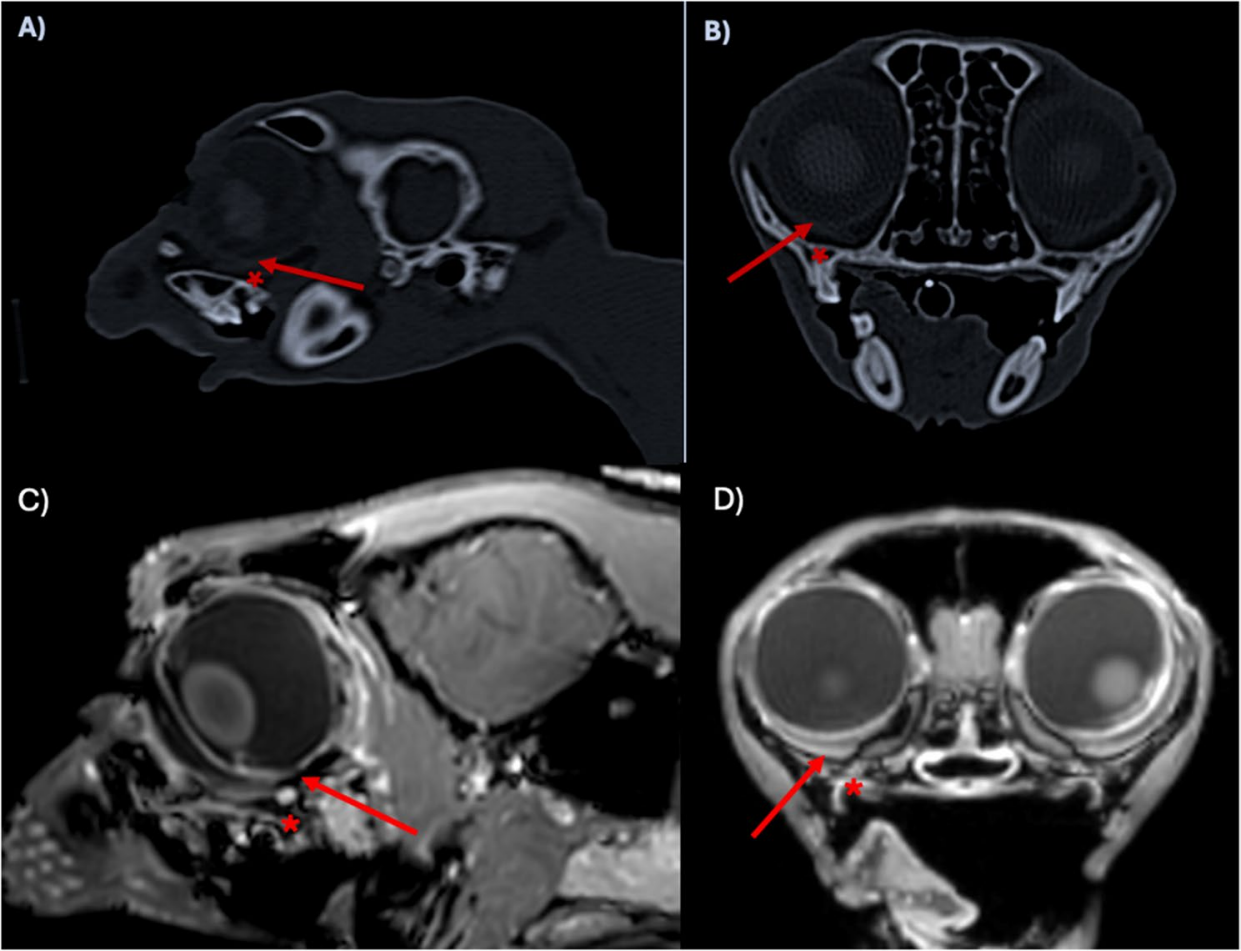
Parasagittal (**A**, **C**) and transverse (**B**, **D**) computed tomography (CT) and magnetic resonance (MR) images of the head of a healthy cat, indicating the proximity of the caudal maxillary tooth roots (red asterisk) and the ventral aspect of the globe (red arrow) to illustrate the thin bony barrier

Although orbital penetration is less frequently reported, the proximity of the caudal maxillary tooth roots and the orbit, combined with the lack of a protective bony barrier in between, makes cats and small breed dogs prone to iatrogenic damage of the globe [1, 3] (Fig. 1).

Complications following dental procedures may occur after maxillary tooth extraction or trauma of the maxillary nerve caused by sharp dental instruments or block of the maxillary nerve [1]. Penetrating ocular trauma frequently leads to panophthalmitis, with first clinical signs occurring within 1–2 days following surgery [1]. In a previous case series in cats with panophthalmitis due to globe penetration during dental procedures, the condition led to enucleation of the globe in about half of the cases [2]. All cats in that case series were referred following extraction of premolar or molar teeth due to ipsilateral ocular changes. The most common ocular findings were anterior chamber fibrin clots, aqueous flare, vision loss, and miosis [2]. The etiology of the panophthalmitis often remains inconclusive. In some cases, only histopathological examination can identify globe penetration and intraocular bacteria, but the exact site of penetration remains undetectable [3].

Although literature specifically describing intracranial complications due to penetrating ocular trauma in cats following dental procedures is limited, in dogs, intracranial complications leading to brain abscessation have been described after a maxillary molar extraction leading to focal fracture of the frontal bone. One dog showed clinical signs of lethargy, loss of appetite, head pressing, epileptic seizure activity, and suffered from exophthalmos. The patient died due to respiratory arrest [4].

Bacterial meningoencephalitis is a rare condition in cats [5]. Described causes include otogenic spread from bacterial otitis media/interna, expansion of inflammatory lesions from the nasal cavity or frontal sinuses, hematogenous spread, or direct infection after bite wounds [6–8]. To date, an ascending bacterial infection of the central nervous system (CNS) via the optic nerve has not yet been reported. The treatment of bacterial meningoencephalitis in cats should be initiated promptly. Medical therapy, surgical intervention, or a combination of both can be used depending on individual cases. In one study comparing different treatment options for intracranial bacterial empyema or abscessation, no statistically significant difference in survival times was found in cats treated with surgery or antibiotics with anti-inflammatory medications alone [9].

Although there is existing literature on potential causes of bacterial meningoencephalitis in cats, ascending bacterial meningoencephalitis following penetrating ocular trauma during a routine dental procedure has not yet been described. This case report presents the clinical signs, examination findings, MRI findings, and necropsy results in a 12-year-old European Shorthair cat presented with swelling of the right eye after a dental procedure.

## Case presentation

A 12-year-old, 5 kg, neutered male domestic short-haired cat was presented to the emergency service of the Small Animal Clinic at the University of Veterinary Medicine Hannover, Germany, with a five-day history of left-sided eye swelling, inappetence, and lethargy.

Previous history included a routine dental procedure during which multiple teeth were extracted at the local veterinarian six days prior to presentation. The day after the procedure, swelling of the left eye was noticed, and the cat had been more lethargic than usual. Additionally, there was a significant reduction in food intake. The owner re-presented the cat to the local veterinarian, where treatment was initiated with systemic anti-inflammatory medication (meloxicam, Boehringer Ingelheim, Ingelheim, Germany, 0.1 mg/kg PO q24h) and topical antibiotic application for the left eye (cloxacillin eye ointment, Zoetis, Parsippany, NJ, USA; frequency unknown). Despite this treatment, the clinical signs progressively worsened over five days; therefore, the cat was referred for further investigations.

**Fig. 2:**
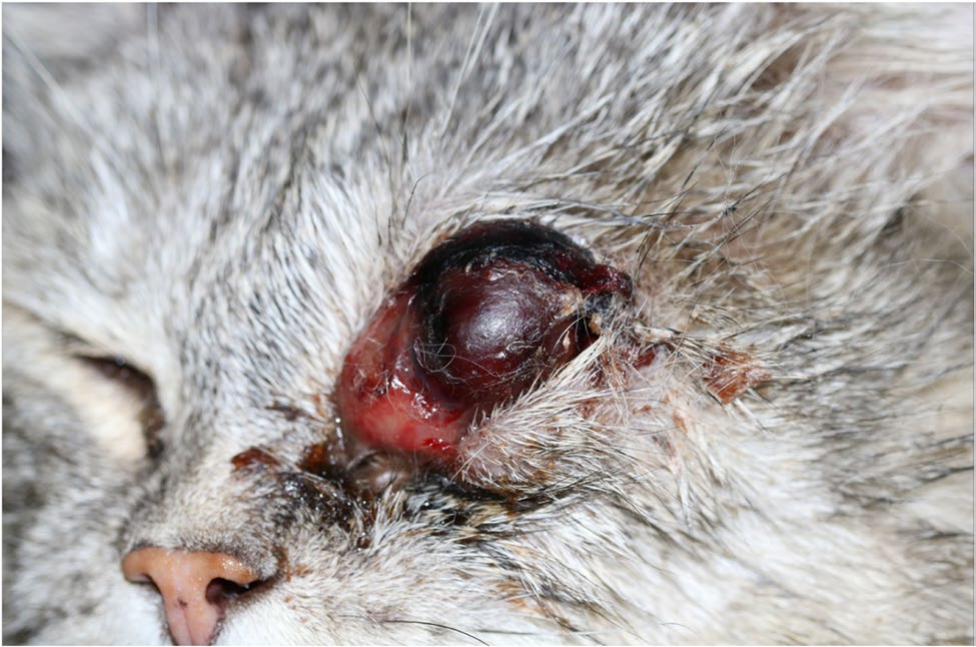
Twelve-year-old male domestic short-haired cat on the day of presentation with marked swelling, exophthalmos, corneal desiccation and hyphema of the left globe

The initial general physical examination revealed marked lethargy, hypersalivation, changes in the oral cavity consistent with erosive gingival lesions, multiple missing teeth (incisors and premolars in the right and left maxilla, premolar in the right mandible), and abnormalities in the left eye (Fig. 2). The left eye was markedly swollen and darkly discolored, indicating hyphema. The conjunctiva was chemotic, and there was mucohemorrhagic discharge. The intraocular structures could not be identified. The rest of the physical examination was unremarkable.

The cat subsequently underwent a complete ophthalmic examination, including neuro-ophthalmological evaluation, Schirmer tear test I reading (Schirmer Tear Test strips; Merck Animal Health; Summit, New Jersey, USA), fluorescein staining (Fluorescein sodium ophthalmic strips USP; Contacare Ophthalmics & Diagnostics; Gujarat, India), rebound tonometry (iCare TONOVET Plus; Icare Finland Oy; Helsinki, Finland), slit-lamp biomicroscopy (SL-17 Portable slit lamp, Kowa; Tokyo, Japan), and funduscopic examination with indirect ophthalmoscopy (Omega 500; Heine Optotechnik; Gilching, Germany).

The right eye had a present menace response and dazzle reflex. The direct pupillary light reflex (PLR) was present, but the indirect PLR elicited by illuminating the contralateral left eye caused no pupillary constriction in the right eye. The right eye had mild iridal hyperpigmentation, suspicious for melanosis. There was mild vitreous prolapse; otherwise, this eye was unremarkable with a normal Schirmer tear test reading and intraocular pressure.

The left eye showed severe abnormalities. Menace response and dazzle reflex were absent. The direct and indirect PLR could not be assessed. Schirmer tear test reading was not performed due to gross macroscopic abnormalities. The periocular region showed marked hemorrhagic and mucoid discharge. Moderate conjunctival hyperemia and chemosis were observed in the medial palpebral and bulbar conjunctiva. There was marked exophthalmos with entrapment of the eyelids behind the equator of the globe. A mild globular protrusion of the cornea was observed, accompanied by severe corneal desiccation. Slit-lamp biomicroscopy revealed a complete hyphema, precluding further examination of the intraocular structures. The intraocular pressure was 99 mmHg (reference: 10–25 mmHg). Due to the severe alterations of the cornea, this measurement is not reliable.

The left eye was non-visual due to complete hyphema formation and severe exophthalmos of unknown etiology. Open-globe injury could not be ruled out, given the altered globe contour and complete hyphema.

 Exophthalmos and hyphema formation were suspected to result from penetrating ocular trauma as a complication of the prior dental procedure. Possible differential diagnoses included traumatic exophthalmos following head injury, intraocular or orbital neoplasia, space-occupying orbital inflammations leading to secondary panophthalmitis, or the presence of an orbital foreign body with secondary destruction of the globe. The changes in the left eye and the oral cavity were suspected to be painful; the cat’s lethargic state and inappetence were also attributed to pain.

Further investigations included hematology, biochemistry, thoracic radiographs, and abdominal ultrasound. Findings revealed leukocytosis of 19.5 x 10³/μl (reference range 6–11 x 10³/μl), mild hyperglycemia (126 mg/dL; reference range 60–100 mg/dL), and hyperproteinemia (9.46 g/dL; reference range 6–8 g/dL).Thoracic radiographs and abdominal ultrasound were unremarkable. Additional diagnostic testing was declined by the owner.

The cat was hospitalized overnight for pain management and received an intravenous continuous-rate infusion of sodium chloride (Sterofundin® ISO, B. Braun Vet Care, Tuttlingen, Germany) at 2 ml/kg/h, combined with fentanyl (Sanacorp Langenhagen, Germany, 1 µg/kg/h) and ketamine (CP-Pharma, Burgdorf, Germany, 0.12 mg/kg/h) until the next morning. Additional treatment included meloxicam (Boehringer Ingelheim, Ingelheim, Germany, 0.05 mg/kg PO q24h), maropitant (CP-Pharma, Burgdorf, Germany, 1 mg/kg IV q24h), and sucralfate (Combustin, Duermentingen, Germany, 1 ml PO q24h). To assess the pain status, a modified Glasgow Coma Scale for cats was applied every three hours. Initially, before the administration of analgesics, the pain score was eight (maximum score: 20). During re-evaluations, the score consistently remained between four and five, and no additional analgesic treatment was added.

Oral exploration under general anesthesia and enucleation of the left eye were scheduled for the following day, for which the cat remained hospitalized.

**Fig. 3:**
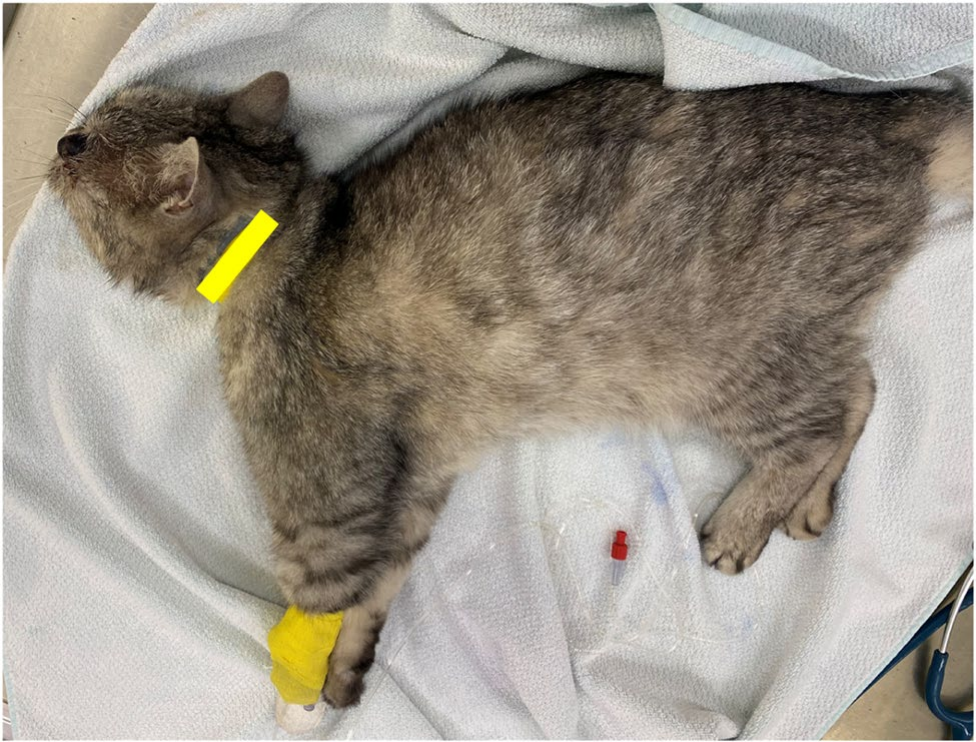
Twelve-year-old male domestic short-haired cat showing clinical deterioration with stupor, opisthotonus, forelimb extension, and hindlimb flexion

On physical examination the next morning, the cat was in lateral recumbency with severely decreased mentation (Fig. 3). On neurological examination, the cat was stuporous and displayed signs of opisthotonus and extension of the thoracic limbs as well as hyperflexion of the pelvic limbs. Cranial nerve examination revealed vertical nystagmus in the right eye, reduced menace response, and intact palpebral reflex and PLR. Examination of the left eye was not possible due to severe swelling and exophthalmos. By the end of the neurological examination, the cat had a generalized tonic-clonic seizure. Therefore, the examination was limited.

The generalized tonic-clonic seizure suggested a corticothalamic neuroanatomical localization, while the nystagmus may have indicated central vestibular system involvement. The stuporous state of consciousness may indicate a diffuse localization involving both the forebrain and brainstem, whereas decerebellate rigidity suggests involvement of the cerebellum. Considering these clinical signs, a multifocal intracranial neuroanatomical localization and increased intracranial pressure, were suspected.

Emergency treatment with intravenous antiseizure medications (diazepam, B. Braun Vet Care, Tuttlingen, Germany, 1 mg/kg IV; phenobarbital, Desitin, Hamburg, Germany, 1 mg/kg IV) and intravenous mannitol (B. Braun Vet Care, Tuttlingen, Germany, 1 g/kg over 20 minutes) were initiated. The owner declined further treatment and diagnostic workup and elected euthanasia.

A postmortem magnetic resonance imaging (MRI) scan of the skull was performed using a 3.0 Tesla MRI scanner (Achieva, Philips Medical Systems, Best, The Netherlands), followed by gross and histopathological examination by a board-certified veterinary pathologist. 

MRI sequences included sagittal, transverse, and dorsal T2-weighted (w) turbo spin-echo (TSE) (TR 5058 ms, TE 19 ms, slice thickness 2.5 mm) images. The MRI revealed an intra-axial, ill-defined, irregularly marginated, oval-shaped mass-like lesion in the thalamus, located slightly caudodorsal to the right optic canal and dorsal to the optic chiasm. The lesion showed heterogeneous signal intensity with a T2w isointense (compared to gray matter) center, surrounded by a broad T2w hyperintense peripheral halo-like rim. The space-occupying lesion caused a significant mass effect with associated deviation and compression of the thalamus, ventricular system, and midbrain, and a mild midline shift to the right side. Moderate caudal transtentorial herniation of both occipital lobes, cerebellar herniation through the foramen magnum, effacement of the cerebral sulci, and mild ventricular dilatation were also observed.

**Fig. 4:**
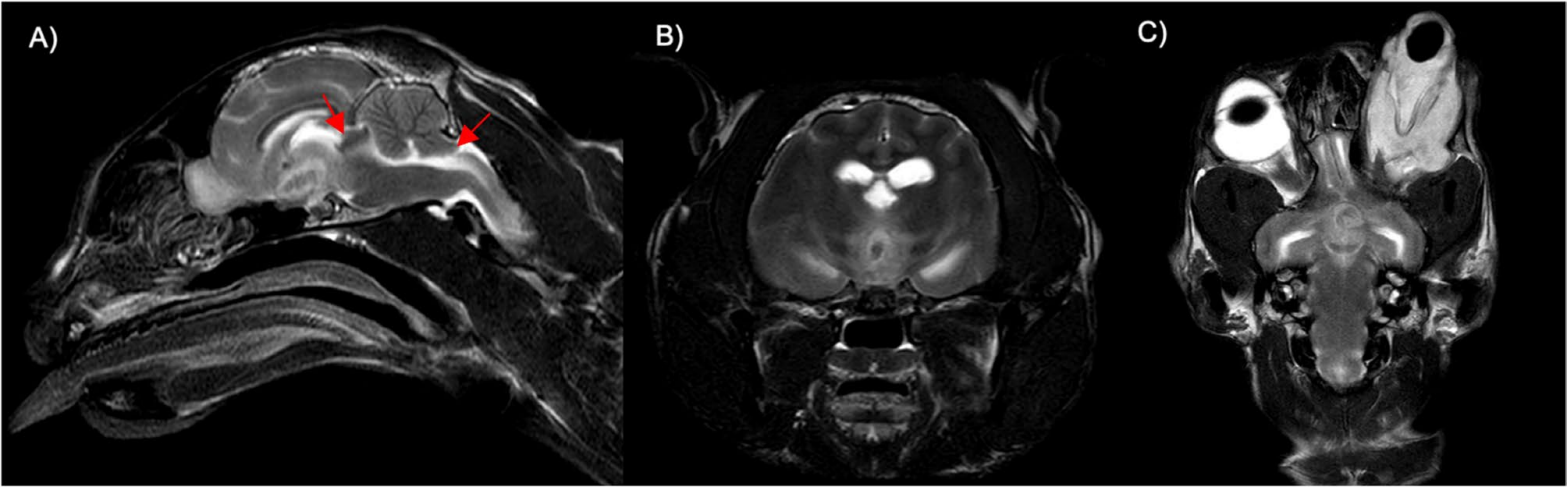
Twelve-year-old male domestic short-haired cat, magnetic resonance imaging (MRI) findings: T2w midsagittal (**A**), transverse (**B**), and dorsal (**C**) MRI images of the brain showing an intra-axial mass lesion in the thalamus with concurrent caudal transtentorial and foramen magnum herniation (indicated by the red arrows) (**A**). A T2w hyperintense (compared to gray matter) ring with hypointense center is present (**B**). There is mild midline shift to the right. (**C**) The left globe extrudes severely out of the orbit and massive retrobulbar accumulation of T2w hyperintense material and loss of definition of extraocular musculature, fat, and the optic nerve is present

In the left orbit, a marked increase in soft tissue volume with T2w hyperintensity (mildly hypointense to cerebrospinal fluid) was present. The left optic nerve was moderately thickened and partially ill-defined and displayed heterogeneously increased signal intensity. Severe loss of definition of the left extraocular muscles and retrobulbar fat was observed. Due to marked extraocular swelling, the globe also showed significant deformation of its contour, with flattening of the anterior chamber and a triangular distortion of the vitreous body. The normally T2w hyperintense fluid in the anterior chamber and vitreous was lost, and a mixed-intensity signal, predominantly T2w hyperintense, was present. The lens was misshaped but in situ. There was no evidence of penetrating trauma to the globe. Findings suggested a retrobulbar mass-like lesion involving the optic nerve, extraocular musculature, and retrobulbar fat, with intracranial extension affecting the optic chiasm (Fig. 4). The findings were consistent with retrobulbar abscess versus orbital cellulitis and optic neuritis. It was presumed that the intracranial lesion represented a brain abscess secondary to the retrobulbar lesion. Based on the presence of caudal transtentorial and foramen magnum herniation, perilesional edema, mass effect of the lesion, distortion of the interthalamic adhesion, and effacement of the cerebral sulci, increased intracranial pressure was suspected.

A full necropsy was performed. Macroscopic findings included severe exophthalmos and deformation of the left eye. Additionally, multiple missing incisors and premolars, as well as a gingival defect with cavity formation in the left and right maxilla in the areas of premolar (P)2 and P3, were observed. Tissue samples, including the brain, spinal cord, left and right eye with retrobulbar tissue, left and right optic nerve, as well as left and right maxillary bone, were fixed in 10% neutral buffered formalin for 24 hours. Transverse sections of the fixed left eye revealed a ruptured retrobulbar abscess surrounding the optic nerve. The brain and spinal cord, including the meninges, were macroscopically unremarkable. After fixation, all tissue samples were routinely processed, embedded in paraffin, and stained with hematoxylin and eosin (H&E) for histopathologic examination.

**Fig. 5:**
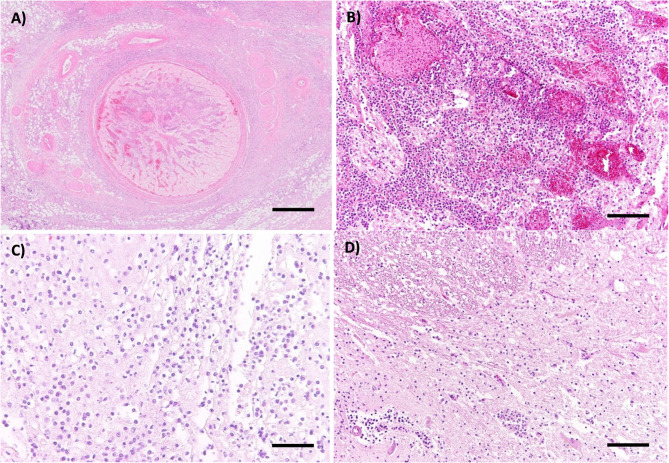
Hematoxylin and eosin stains of the left optic nerve (**A**, **B**), brainstem (**C**), and spinal cord (**D**). **A**) Cross section of the left optic nerve. The overview shows marked hypercellularity within the nerve as well as the perineural tissue. Scale bar = 500 µm **B**) Higher magnification reveals severe, multifocal to coalescing, suppurative and necrotizing neuritis and multifocal hemorrhages. Scale bar = 100 µm **C**) Moderate lymphoplasmacytic, partly neutrophilic inflammation of the brainstem. Scale bar = 50 µm **D**) Mild to moderate lymphoplasmacytic, partly neutrophilic inflammation of the spinal cord. Scale bar = 100 µm

Besides the macroscopically evident retrobulbar abscess, H&E stains revealed a severe, multifocal to coalescing, purulent and necrotizing panophthalmitis of the left eye, along with neuritis of the left optic nerve with intralesional bacterial colonies and formation of granulation tissue. Lymphoplasmacytic to lymphohistiocytic and partially purulent inflammation was also found to varying degrees in the cerebellum, brainstem, cerebrum, spinal cord, meninges, as well as in the right eye and right optic nerve, although no abscess formation was detected in these areas (Fig. 5).

**Fig. 6:**
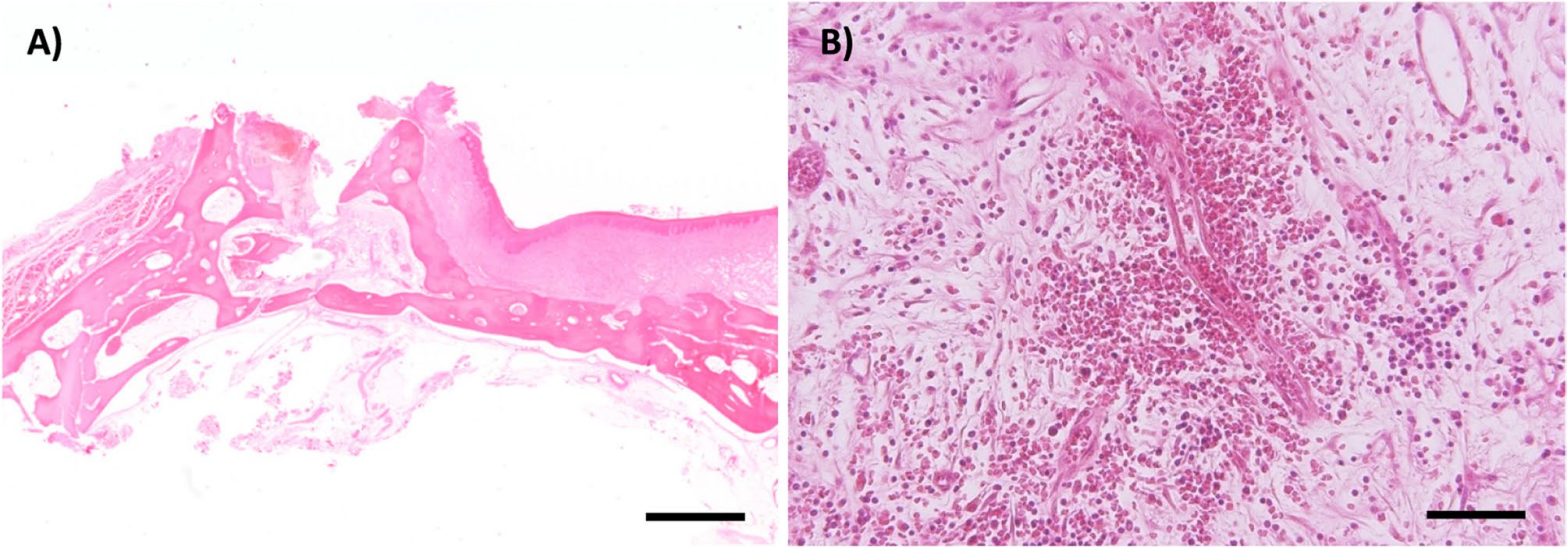
Hematoxylin and eosin stains of the upper jaw. **A**) Tooth cavity of the left upper P2. Scale bar = 2 mm **B**) Higher magnification reveals a focal, moderate hemorrhage, mild lymphoplasmacytic and suppurative inflammation with formation of granulation tissue. Scale bar = 50 µm

The altered areas in the left and right maxillae at the level of the P2 and P3 showed moderate, focal bleeding and granulation tissue formation. In the left maxilla, a mild to moderate lymphoplasmacytic, partially purulent inflammation was present (Fig. 6).

A swab sample was taken from the left retrobulbar space (aerobic and anaerobic) and cultured moderate levels of *Peptostreptococcus canis*. Under aerobic conditions, no bacterial growth was detected.

## Discussion and conclusion

This case report describes a rare but severe complication in a 12-year-old domestic short-haired cat following a routine dental procedure, leading to retrobulbar abscessation and presumed ascending bacterial meningoencephalitis.

Although such complications are rare, early signs such as lethargy, inappetence, and exophthalmos may be indicative of this condition. This is consistent with previous reports in cats, where clinical signs typically developed within an average of one and a half days following tooth extraction, as observed in our patient [2].

 Despite medical treatment focused on the ocular changes, the clinical condition deteriorated rapidly. Within one day, signs of intracranial involvement were present, which had not been apparent initially. The cat was subsequently euthanized at the owner’s request due to the rapid and severe deterioration.

Postmortem MRI revealed an intra-axial T2w hyperintense lesion in the thalamus with concurrent caudal transtentorial and foramen magnum herniation. An intra-axial T2w hyperintense signal with pronounced mass effect is common in the acute phase of abscess formation [8, 10], which is comparable to the postmortem MRI images obtained in the present case. However, one limitation in this case is that no post-contrast images could be obtained on MRI, which might have demonstrated the typically described ring enhancement of an abscess capsule [8, 10]. Although these sequences could not be obtained due to the postmortem nature of the scan, the imaging findings suggested an abscess in the thalamus. Significant retrobulbar soft tissue changes and left globe protrusion were also noted. Considering the ocular and retrobulbar changes and involvement of the optic nerve, retrobulbar abscess formation and ascending optic neuritis leading to bacterial meningoencephalitis was highly suspected after MRI.

 Necropsy confirmed clinical and imaging findings such as inflammatory gingival lesions, bacterial panophthalmitis, optic neuritis with retrobulbar abscess formation, and severe meningoencephalitis. However, no macroscopically visible abscess with a capsule, as suspected based on imaging, was found. Bacterial infection was proven by microbiological culture from the retrobulbar space revealing the presence of*Peptostreptococcus canis*, a gram-positive, obligate anaerobic organism, previously identified in cats with periodontal disease, as well as in subgingival plaques of Labrador Retrievers [10–13]. This pathogen was also described in a human case report, where it was isolated from a periprosthetic joint infection following wound contact with dog saliva. This patient received penicillin and rifampicin [15]. However, to treat this pathogen, beta-lactam antibiotics are recommended in the human literature [15]. In veterinary medicine, penetration of the blood-brain barrier can be achieved with beta-lactam antibiotics, and prompt treatment could have been beneficial in the described case [14].

Isolated pathogens when investigating orbital inflammation in cats included*Pasteurella species, Bacteroides species, and Actinomyces species* [9, 11]. Of the cases studied, 60% involved purely aerobic bacterial infections, while mixed aerobic and anaerobic bacteria were cultured in 20% of the cases. In a previous case series of cats that developed ocular complications following dental treatment, no pathogens were isolated from the examined globes. However, all cats were treated with antibiotics, which included but were not limited to cefovecin, amoxicillin-clavulanic acid, and doxycycline [2]. No case of clinical deterioration suggestive of intracranial involvement was reported in this study. It may be assumed that the early recognition and initiation of antibiotic treatment of the panophthalmitis might prevent further spread from the eye and thereby improve the case outcome.

The histopathological examination of the left globe revealed a suppurative infection, consistent with panophthalmitis [3]. With regard to the etiology of the inflammation, no clear puncture site could be identified in the current case, although such sites have been reported in some cases in the literature [2]. It remains unclear whether the primary injury was due to maxillary tooth extraction using sharp dental instruments or from maxillary nerve block. Both traumatic globe penetration and primary retrobulbar infection, with secondary bacterial contamination of the globe causing severe panophthalmitis, are plausible [1].

Retrobulbar abscesses, as described in this case, are a recognized complication of ocular trauma from dental procedures and a possible cause of secondary exophthalmos [12]. In the described case, however, there was severe meningeal inflammation adjacent to the optic chiasm. Taking all the diagnostic findings into consideration, an ascending bacterial infection via the oral lesion to the globe and retrobulbar space, and via the optic nerve to the optic chiasm and into the brain, is most likely the pathogenesis in the present case. Signs of purulent inflammation could be detected on MRI and histopathology. A hematogenous spread of infection cannot be completely excluded but seems unlikely, since the inflammation of the whole optic nerve could be well identified. For cases of orbital trauma and panophthalmitis with suspected brain involvement, advanced diagnostic imaging and collection of cerebrospinal fluid for cell count and microbiological culture are recommended (unless there is increased intracranial pressure). Cats with ocular changes should be hospitalized early, since rapid deterioration due to ascending inflammation can result in life-threatening conditions. Early administration of an antibiotic that crosses the blood-brain barrier should be considered for treatment. Pharmacokinetic data on CNS-penetrating antibiotics in healthy cats or cats with inflammatory brain conditions are still incomplete, and uniform treatment guidelines do not exist. Possible options discussed include, for example, clindamycin, benzylpenicillin, beta-lactams, fluoroquinolones, trimethoprim-sulfadiazine, and metronidazole [14]. Some authors alsodiscuss the short-term use of corticosteroids prior to antibioticadministration to reduce the inflammatory response to dying microorganisms [7, 15, 17].

There are several limitations in this case report. At the time of presentation, limited information was available regarding the patient’s previous history. A surgical report on the tooth extractions and detailed post-operative findings were not provided by the referring veterinarian. Additionally, cerebrospinal fluid (CSF) analysis or blood cultures were not performed, since the cat was humanely euthanized at the request of the owner. Postmortem CSF sampling might have been possible, but samples for bacterial culturing were only taken in the necropsy hall. In addition, due to the postmortem nature of the study, no contrast study could be performed on MRI. Sadly, the specific anatomical connection between the oral cavity and the orbit could not be confirmed, likely due to the very small lesion (possibly caused by a puncture incision). An abscess suspected on MRI could not be confirmed in the histopathological examination. The T2 hyperintense areas may have represented edema, while the hypointense center might also indicate normal grey matter instead of necrosis. Although a full correlation between MRI findings and pathological results was lacking, the MRI still demonstrated extraocular and intracranial changes as well as signs of increased intracranial pressure.

To our knowledge, this is the first reported case in a cat suffering from panophthalmitis, retrobulbar abscess formation and bacterial meningoencephalitis with secondary increased intracranial pressure as a complication following a dental procedure and resulting in poor outcome.

In conclusion, cats presenting with ocular changes after dental treatment should be closely monitored and treated early, as rapid deterioration due to ascending inflammation can be life-threatening. Ascending bacterial optic neuritis and meningoencephalitis are rare but possible complications after dental procedures, necessitating early detection and intervention for effective treatment.

## Data Availability

The raw data supporting the conclusions of this article will be made available by the authors, without undue reservation.

## Abbreviations

CNS: Central nervous system
CT: Computed tomography
Fig.: Figure
H&E: Hematoxylin and eosin
IV: Intra venous
MRI: Magnetic resonance imaging
P: Premolar
PLR: Pupillary light reflex
PO: Per os
w: Weighted
